# Guy's Hospital Reports

**Published:** 1840-04

**Authors:** 


### Art. IV.

*Guy's Hospital Reports.* Nos. VIII.-IX. *April and October*, 1839.—*London.* 8vo, pp. 448.

The successive numbers of this publication keep up the character of general excellence, which we have more than once had the pleasure of assigning to it. On the present occasion, as formerly, we shall best evince our sense of its value by giving a detailed account of its principal contents.

### No. VIII.

1. *On the Disorders of the Brain connected with Diseased Kidneys.* By Dr. Addison.—The object of this communication is, according to its author, threefold: "First, to point out the general character and individual forms of cerebral disorder connected with interrupted function of the kidneys, from whatever cause such interruption may arise. Secondly, to show that, in recent as well as in chronic disease of the kidney, the cerebral disease is not unfrequently the most prominent, and occasionally the only obvious symptom present. And, thirdly, to establish a means of diagnosis, in such obscure or in unsuspected cases, upon the peculiar character of the cerebral affection."

It seems hardly fair to deal harshly with an author who assures us that the details of his production are imperfect, and his aim rather to excite enquiry, than to promulgate truths, which he presumes he has established: nevertheless we cannot forbear asking Dr. Addison, why—with the consciousness that his essay is deficient, deficient in the most essential particular, proofs of the novel dogma it advances,—he has shown himself so anxious to lay it before the public? Why present the profession with "imaginings" and "beliefs," instead of patiently waiting, until he could substantiate these by the evidence of closely observed and faithfully related cases? Dr. Addison, however, was at liberty to choose his own method of proceeding; let us enquire into its results.

The general character of cerebral affections connected with renal disease is believed by this writer to be "marked by a pale face, a quiet pulse, a contracted or undilated and obedient pupil, and the absence of paralysis;" and the individual forms of this cerebral disorder are stated to be the five following: "1. A more or less sudden attack of *quiet stupor;* which may be temporary and repeated, or permanent, ending in death. 2. A sudden attack of a *peculiar modification of coma and stertor;* which may be temporary or end in death. 3. A sudden attack of *convulsions;* which may be temporary or end in death. 4. *A combination of the two latter;* consisting of a sudden attack of coma and stertor, accompanied by constant or intermitting convulsions. 5. A state of *dulness of intellect, sluggishness of manner and drowsiness,* often preceded by *giddiness, dimness of sight, and pain in the head,* proceeding either to *coma* alone, or to *coma accompanied by convulsions;* the coma presenting the peculiar characters already alluded to."

We really can perceive nothing novel in this enumeration, except the style of classification adopted: there is not a single statement of the least importance in Dr. Addison's account of these five cerebral conditions, of which the substance may not be found clearly and explicitly laid down in Dr. Christison's recent treatise on Bright's disease. Of these forms of cerebral disorder, with which all experienced observers are perfectly well acquainted, we expected no illustrative cases at the hands of our author; but we do require him to bring forward a good supply to demonstrate to us what he asserts, and what the essay putatively "*shows*" to be the fact, that cerebral symptoms will sometimes lead to the diagnosis of renal disease, when every ordinary symptom of such affection is absent. The practical bearing of this proposition is manifest and important; but almost the sole evidence adduced in its support, consists of the following loose statement, for which Dr. Addison seems to have trusted to his memory alone: "the most exquisite example I ever saw of the state of quiet torpor occurred in a man who at the time presented no dropsical symptom, whose urine was not albuminous, and who made no complaint of pain or uneasiness in his loins." The man's perception of pain was of course blunted by the state of his cerebral functions, but was no suffering evinced under forcible and long-continued pressure in the renal regions? What were the physical and chemical characters of the urine, as detected with and without the aid of the microscope? Was there vomiting or not? And above all, admitting that an enquiry into these particulars is liable to be neglected in the hurry of practice, and that a peculiar state of the brain would be likely to arouse the attention of even a very careless practitioner, in what proportion of cases does Dr. Addison affirm that the ordinary symptoms of nephritis are supplanted or masked by this disturbance of the brain, for upon this ultimately depends the bedside utility of the alleged discovery? If, as we strongly suspect, such cases are of most extraordinarily rare occurrence, (we doubt if they can *ever* occur in the practice of the philosophic observer, who studiously examines the condition of every organ, whether presumed to be diseased or not,) there would be no little temerity in grounding a diagnosis on what must be termed a bare possibility. Be this as it may, "the cortical part of the kidneys was found highly injected, of a deep red or almost chocolate colour, and somewhat softened in its texture," in the subject in question.

This observer purposely omits to notice the morbid changes discovered in the brain after death, because "they are well known to be very often, in appearance at least, extremely slight." We should have considered the prevalence of this opinion respecting the unaltered condition of the cerebral tissue a strong motive for adopting an opposite line of conduct: if the general impression be a wrong one it should be corrected; if the contrary, the greater the mass of evidence of its justness we have the better, for if there be a science in existence, in which negative facts occasionally acquire the full weight and importance of positive ones, that science is pathology.

II. *On Perforations of the Stomach from Poisoning and Disease.* By Mr. Alfred S. Taylor.—Mr. Taylor here endeavours to establish the distinctive signs of perforation of the stomach, when caused by chronic disease of its coats, and when resulting from the action of poisons, with greater precision than had hitherto been attained. And his efforts have been far from unsuccessful; with but little really novel matter to assist him, he has, by his excellent arrangement of previously ascertained facts, his close investigation and shrewd inferences from their details, managed to place the whole subject in a clearer point of view than any of his predecessors. The nature of the essay, however, founded as it is for the most part on materials with which the reader of modern works on forensic medicine is familiar, will prevent us from analyzing it at any length: a sketch of its plan, and an occasional notice of some of the arguments with which it is interspersed, will, we are persuaded, give the reader a desire to become acquainted with the original.

Mr. Taylor enumerates four cases in legal medicine, wherein a knowledge of this subject will be required of a medical practitioner: "1. A person may have died from perforation of the stomach through disease and not from poison. 2. A person labouring under the disease may be the subject of poison. 3. A person labouring under the disease may have received blows or injuries on the abdomen; in which case it will be necessary to state whether the perforation did or did not result from the violence used. 4. Perforation of the stomach from post-mortem changes may be mistaken for perforation from poison." The author opens his investigation of these cases by successively considering the peculiarities of perforation caused by *poisons*, by *morbid causes*, and by *post-mortem changes*.

Perforation of the stomach from natural causes, that is, from ulceration, simple or scirrhous, and from solution of the parietes of the organ, involves no few points yet open to debate. These, however, rather belong to perforation from the latter cause, than to that originating in ulceration. Indeed, the characters of this insidious and most formidable morbid process are so well defined in the vast majority of cases, both by the almost total absence of important symptoms, until perforation is actually accomplished, and by the anatomical peculiarities of the opening, that there has been very little scope for variance in the descriptions of authors. From the cases collected by Mr. Taylor, the following conclusions clearly derive: Ulcerative perforation seems most frequently to occur in young females from eighteen to twenty-three years of age, but has been observed in subjects of advanced age and of both sexes. The preceding illness rarely amounts to more than simple dyspepsia, or slight irritation of the stomach after eating, with want of appetite. These ailments may have existed for weeks or months, without exciting much attention on the part of the patient, when she is seized with excruciating pain in the abdomen, (generally, but not always, soon after a meal,) in the majority of cases accompanied with pain between the shoulders. Vomiting commonly ensues, but not invariably; there is no diarrhœa; on the contrary, obstinate constipation is usually observed: with the progress of the disorder the symptoms become distinctly those of peritonitis, death supervening in from eighteen to thirty-six hours. Mr. Taylor's cases are defectively reported in respect of the condition of the pulse in the early stage of the abdominal pain; if the statement of Mr. Travers (*Med. Chir. Trans.* vol. viii.), to the effect that its frequency remains natural for some time, until the symptoms merge in those of peritonitis, be correct, the diagnostic importance of the state of arterial pulsation needs not to be insisted on.

It will be readily admitted that there is quite enough in the character and course of the symptoms enumerated to excite the suspicion of poisoning, which almost always arises in the minds of non-professional persons, when death occurs under circumstances of the kind. The previous good health of the patient, the seat of the pain, its appearance soon after eating, and the suddenness of dissolution form a strong chain of evidence against the arguments of the practitioner, who is shrewd enough to suspect the true nature of the case. The post-mortem appearances—a characteristic aperture in the stomach, the presence of the contents of that organ in the cavity of the peritoneum, and the marks of recent peritonitis—will convert his suspicion into certainty. The aperture is commonly of an oval or rounded form, from half an inch to an inch in diameter, and almost invariably situated in or near the lesser curvature between the cardia and pylorus. The characters of the margin vary, according as the ulceration is scirrhous or simple. In the former case the edges are smooth and fleshy-looking; the tunics of the organ are bevelled off from within outwards; there is no appearance of laceration in the peritoneal coat; near the circumference of the aperture the tissues of the stomach are thickened and indurated, the thickened part forming a narrow ring around it, or extending to some distance over the surface of the organ. In cases of simple ulcerative perforation there is thickening, and marks of ulceration are apparent; the edge of the opening may be very slightly or not at all raised above the surface of the surrounding membrane. Such is Mr. Taylor's description, from actual observation, of this very important lesion; his experience closely agrees with that of other observers.

It is difficult to understand how it happens that marks of laceration are not usually found in the perforated peritoneum: Mr. Taylor's belief that this membrane is reduced by slow absorption to the thinnest possible stratum, before the opening is actually formed, in nowise removes the difficulty; for, admitting the conjecture to be just, it would merely explain excessive delicacy, but certainly not absence of the fringed detritus, which *à priori* views would lead us to look for. The resistance to the ulcerative process offered in some instances by the peritoneum is well illustrated by a remarkable and, we believe, unique case recently published by Mr. Crowfoot. (*Lancet*, January, 1839.) The patient died in thirty hours after the supervention of well-marked symptoms of perforated stomach: six months previously she had had an attack of profuse hematemesis, and in the interim been subject to "stomach attacks." The mucous membrane of the stomach was found healthy; the perforation, about the size of a sixpence, had only traversed the muscular and mucous coats, and led to a cavity almost equal to the stomach in size, lined with a pseudo-mucous membrane, and bounded by the peritoneal and muscular coats of the organ. Several pin-hole openings were discovered in the peritoneal portion of this pouch; through these the effusion of the contents of the stomach had taken place. Now it is exceedingly probable that, at the period when the patient was seized with hematemesis, ulceration through the two internal tunics took place, and that the formation of the cavity between these and the external coat then commenced. The case curiously corroborates the observations made by Professor Sebastian, of Groningen, on a similar pouch-like dilatation of the investing peritoneum consequent on perforation of the intestine. (*Br. and For. Med. Rev.*, Vol. VII. p. 248.)

Perforation by corrosive poisons, such as sulphuric acid, is certainly not likely to be confounded with the lesion just described; we refer to Mr. Taylor's pages for their distinctive characters. But the case may be different, when the destruction of the walls of the stomach proceeds from the action of certain irritants, such as arsenious acid and the chloride of barium: fortunately, these substances very rarely induce perforation. In making our diagnosis, when doubt arises, we must be guided by the relative frequency of the two kinds of perforation, the period at which the symptoms appear, the character and degree of suddenness of the pain, the absence or presence of vomiting and diarrhœa, the time at which death supervenes, the anatomical state of the peritoneum and of the mucous coat of the alimentary passages, the appearance of the aperture, the results of analysis of the contents of the stomach, and of the matters, if any, vomited during life. In considering these various points, Mr. Taylor most ingeniously avails himself of his intimate acquaintance with the phenomena of arsenical poisoning: he is occasionally speculative, but justifiably so, for in truth the extremely limited number of cases of total destruction of the coats of the stomach by arsenious acid on record (there are only three well-authenticated instances of such an occurrence), makes it impossible to draw decisive inferences in every instance from past experience; and legal medicine is a *science of action.*

There is one statement in this part of the essay with which we cannot agree: Mr. Taylor observes, "when the deceased has taken food alone, chemical evidence cannot be dispensed with, but when in company with others, the simple fact of no other but the deceased having afterwards suffered will go to disprove the fact of poisoning." It is manifest that it will do no such thing, for one of the party may have been the murderer, and will have taken care either to feign eating, if the whole dish be poisoned, or simply to supply the plate of his victim with the poison. And, again, we affirm that there is no possible combination of circumstances, where a suspicion of poisoning has, on any grounds whatsoever, arisen, in which it is not imperative on the practitioner to take measures for the thorough analysis of the products of vomiting, and of the contents of the alimentary canal.

It frequently becomes a matter of serious moment, medico-legally speaking, to determine whether acts of volition and locomotion are possible after the occurrence of certain spontaneous traumatic lesions. In the instance of perforation we can conceive such a question being raised, and Mr. Taylor answers it by anticipation in the affirmative, on the evidence of a case in which it was clearly ascertained by the attendant surgeon that the patient walked to her home, a distance of a quarter of a mile, after the first alarming symptoms had manifested themselves. Disturbance of the intellect is not mentioned among the symptoms in any case, and in some the cerebral functions appear to have maintained their integrity to the last.

We have briefly alluded to the trifling derangement of the digestive functions, which ordinarily forms the only symptomatic evidence, that the process of ulceration is going on in the stomach; but it would even appear, judging from an extraordinary case related by Dr. J. Beck, that actual perforation may take place, unannounced either by pain or any of its usual symptoms, unless the contents of the organ be effused into the peritoneum. The subject of this case, a female, aged sixty, is said to have died evidently from disease of the chest, and never to have complained of uneasiness in the stomach: the mucous membrane of the latter viscus was found in a highly vascular condition, and there were four ulcers, besides the aperture, along the lesser curvature; the aperture was about the size of a shilling. The stomach contained about a pint of fluid; none of its contents had escaped into the peritoneal cavity.

Mr. Taylor's disquisition on perforation of the stomach by solution is sagaciously sustained, and shows him to be possessed of accurate pathological knowledge and considerable talent for close reasoning. Some of the opinions current among the profession on this subject are shown to be of very questionable correctness, and the experiments of Müller and Schwann, with pieces of dried stomach immersed in artificial acidulous mixtures sensibly rejected, as incapable of furnishing any just inference on doubtful points in the history of perforation by the gastric juice. We cannot follow our author through this section of his essay, but subjoin his conclusions.

"1. Perforations of the stomach from solution are very rare in the human subject. 2. They may occur in healthy and diseased states of that body. 3. The perforation takes place after death, and depends on the action of the gastric juice, which, in the opinion of some, is facilitated by a diseased state of the parietes of the stomach. But that the secretion is the chief, if not the sole cause, seems probable, from the fact that the liver, spleen, and diaphragm have also been found softened, the latter even perforated where lying near the aperture in the stomach. 4. The secretion cannot be the healthy gastric-juice, but some altered state of that liquid. This is rendered probable by the facts—*a.* if it were the ordinary secretion, perforation would be much more common in healthy persons, dying suddenly, soon after a meal; *b.* it would not be met with in diseased subjects, or those labouring under disease of the stomach. 5. The exact nature of the liquid producing the change, and the circumstances to which it owes its solvent power, are unknown."

Mr. Taylor having thus laid his foundation for an examination of the medico-legal questions, which we have already transcribed, proceeds to illustrate these by reference to cases. Death from ulcerative perforation is exemplified by the case of a young woman observed last year by Mr. Hilton and himself; the description of the stomach is exceedingly well done, and the form and situation of the aperture shown in an engraving.

A person labouring under the disease may, we have said, be the subject of poisoning: of this, which may be regarded as a rare coincidence, there are two recent examples on record. The principal question here is whether the poison caused the symptoms and death; the fact of its having been swallowed is not supposed to be disputed. The first case referred to is that, related by Wildberg, of a woman, who swallowed by mistake half an ounce of hydrochlorate of baryta, and died in convulsions two hours after; the second that of a man to whom arsenic was administered with his dinner by his wife. In the former instance perforation, with the majority of the characters assigned to the species resulting from chronic disease, existed in the stomach; but so imperfect is the report of the case, that the state of the peritoneum even is not noted.

It is therefore impossible to affirm positively that perforation was produced by disease, but, from the nature of the symptoms, no doubt could be entertained of death having been caused by the poison. In the arsenical case, a scirrhous ulcer, evidently of long standing, was discovered in the stomach near the pylorus, but there was no perforation: it was contended by the prisoner's counsel on the trial, which took place about two years since on the Western Circuit, that the symptoms and their fatal termination were owing to this chronic disease and not to poisoning. But the course of the symptoms, as well as the lesions detected in the stomach, clearly announced the action of some powerful irritant, and this, combined with the fact that death was very unlikely to have occurred from mere ulceration of the stomach without perforation, decided the medical witnesses in giving it as their opinion, that life had been destroyed by the arsenic swallowed.

The third question, respecting the influence of external violence on perforation, receives very imperfect illustration from a wretchedly reported case by Mr. Watson.

Perforation of the stomach from post-mortem changes may be mistaken for that produced by poison, when symptoms, resembling those of irritant poisoning have been observed during life, though no poison has really been administered. Such a combination of circumstances must be singularly unusual. "This, however," observes Mr. Taylor, "signifies little in a legal point of view, for persons charged with crimes are frequently acquitted on the barest medical possibilities." The notorious case of Miss Burns, for whose supposed murder a Mr. Angus was tried a good many years since at Liverpool, is adverted to, in proof of such a difficult point as that just stated being occasionally submitted to the judgment of the medical witness. The case is, however, not examined at length, and for this very excellent reason, that the reports of the post-mortem appearances are imperfect.

We recommend this excellent paper to the notice of all our readers.

III. *On the Diurnal Variations of the Pulse.* By Dr. W. A. Guy.—This is a continuation of the author's previous enquiries, and is marked by the same clear and philosophical views. The inferences drawn by Dr. Guy, respecting the diurnal changes in the frequency of arterial pulsation, from a series of experiments conducted on his own person, are no doubt applicable to the future healthy action of his own pulse. But it may be questioned (as the experimentalist himself admits), to what extent these observations, made on a single individual, allow of general application. Dr. Guy urges, in support of their catholic pretensions, that he enjoys most excellent health, and has never suffered from any disease affecting the organs of circulation; that his pulse beats with, as nearly as possible, the same average frequency, as that of such healthy male adults as he has had an opportunity of comparing it with, and is affected to a similar extent by change of posture as theirs. Whether these arguments be convincing or not, no one will, we imagine, be captious enough to deny that the results of Dr. Guy's patient and carefully performed experiments are worthy of incomparably greater confidence than the vague impressions on the subject with which medical men have been hitherto satisfied. For our parts, we declare our determination to abide by the following propositions, until a series of equally accurate experiments on a more extended scale shall have shown that they do not in all cases hold good.

"1. The pulse of a healthy male adult in a state of rest, unexcited either by food or exercise, is most frequent in the morning, and gradually diminishes as the day advances. 2. The pulse diminishes in frequency more rapidly in the evening than in the morning. 3. The diminution of the frequency of the pulse is more regular and progressive in the evening than in the morning. 4. The effect of food is greater and more lasting in the morning than in the evening; and in some instances the same food which in the morning produces an effect considerable, both in amount and in duration, has no effect whatever in the evening."

IV. *Observations on Poisoning by the Vapours of Burning Charcoal and Coals.* By Dr. Golding Bird.—If Dr. Bird's essay can lay claim to no other merit, it at least possesses that of seasonableness: the recent exposure of ignorance respecting the most obvious and invariable morbid appearances after death from the inhalation of charcoal fumes, certainly rendered the want of some clearer, fuller, and more methodized account of this description of poisoning, than those to be found in existing works, painfully evident. Dr. Bird has, accordingly, if we may be permitted to be poetical, "seized fleet occasion by the hair," and shown himself particularly anxious to improve the state of his brethren's knowledge with the least possible outlay of time. But it would perhaps have been as well had Dr. Bird recollected that the race is not always to the swift; at least it can hardly be doubted that if he had extended his researches in periodical works, and allowed himself time for the performance of a greater number, and especially of more important experiments, than those he has engaged in, he would have produced a paper, not only more creditable to himself, but one more likely to put an early period to the perplexities of medical witnesses, than the compilation we are about to notice.

It has been remarked by Sachs, as we learn from Dr. Bird, that "what we most accurately know of carbonaceous vapour is the fact of its deleterious nature; but what this really is, or which is the poisonous ingredient, we really do not know." This if not a cheering is certainly a correct estimate of our knowledge on the subject. Let us consider for a moment the number and contradictory character of opinions held on the nature of the toxic principles of charcoal fumes, and the truth of this will become apparent. The mass of practitioners assuming that, what, they fancy, *ought to be*, actually *is*, believe carbonic acid to be the sole destructive principle evolved: but it is alleged that deleterious effects have been produced by charcoal stoves, when the proportion of carbonic acid generated must have been so trifling as to be incapable of accounting for those effects; besides the fallacy of this notion has, it is said, been still more conclusively proved by the fact, that after every trace of free carbonic acid has been removed by lime water from an apartment, in which individuals have been found in a lifeless condition, others on entering it have experienced the usual symptoms of poisoning by charcoal vapour. Hunefield affirms that the poison consists of a volatile mixture of empyreumatic oil, resin, naphthalin, and pyro-carbonic acid; Dr. Bird is quite certain that this result should be considered to refer to wood-smoke—rather a positive mode of deciding the matter, when, as he admits, he has not seen the German Professor's work, and the term employed in the quotation he has met with (*kohlendunst*,) is not, so far as we can ascertain, open to such interpretation. Sachs conjectures that the poisonous ingredient may consist merely of carbon, suspended in a pulverulent form in the air and aspired into the air-passages: this hypothesis is unsupported by experimental proof. Again, Dr. Bird, reflecting on the property possessed by charcoal of absorbing gases and aqueous vapour by exposure to the atmosphere, became anxious to determine whether charcoal might not, when heated in the air, evolve cyanogen or some of its compounds, as moist charcoal saturated with air contains all the elements for the formation of hydrocyanic acid: an experiment was accordingly instituted, but without leading to the detection of any of the acid. It is but just to state, especially as Dr. Bird has not himself done so, that Mr. Coathupe, of Bristol, some time since announced in the Lancet (Jan. 1839), that he had discovered in the peculiar volatile product of burning charcoal, which, according to him, constitutes its really toxic ingredient, a body closely resembling cyanogen in most of its properties. Finally, though last, assuredly not least, Berzelius expresses himself as follows: "Charcoal fume consists neither of carbonic acid nor oxide, nor of light carburetted hydrogen, but of a peculiar combustible body, that soon produces vertigo, headach, vomiting, and even death, notwithstanding that sufficient oxygen may be present in the apartment to support animal life." The analysis of Orfila of the products of dimly burning charcoal, and of the same substance in a state of active combustion, as well as the support they lend to Dr. Babington's hypothesis, respecting the agency of carburetted hydrogen in producing the noxious influence of charcoal vapour, are too well known to justify more lengthened mention here.

Curiously enough, Dr. Bird's conclusion on the subject is "that carbonic acid obviously forms the most important ingredient in the products of charcoal combustion:" elsewhere he goes further, indeed, and forgetful of, or at least indifferent to, opinions emanating even from Berzelius, he rather dogmatically terms the gas in question "*the* poisonous ingredient of charcoal vapour." That carburetted hydrogen is not the destructive principle of the fumes of dimly burning charcoal appears, according to Dr. Bird, to be proved by the fact that it is frequently present in coal mines in explosive proportion, without causing mischief to the miners respiring it. But this is an unsound argument, for, as we shall have occasion to mention in noticing the next paper, most serious consequences have been known to ensue from the inhalation of an atmosphere impregnated with coal gas, though not to a sufficient amount to admit of explosion. Dr. Bird suggests that the more deleterious influence of the fumes of slowly burning charcoal in comparison with those of the same fuel in a state of vivid combustion, should be ascribed to the greater quantity of heat evolved in the latter instance, whereby stagnation of the carbonic acid generated is more effectually prevented.

Having settled the point at issue in his own way, our author proceeds to enquire whether the carbonic acid, evolved from a burning chauffer in an unventilated apartment, descends towards the floor or mounts towards the ceiling. Its specific gravity carries it down; its elevated temperature tends to raise it to the upper strata of air; the law of the mutual diffusion of gases, to disperse it equably through the apartment. The proportional influence of each of these agencies admits of calculation, but the result of direct analysis of the air at different heights is a method of deciding the question less open to cavil. Now, Dr. Bird declares, "he has had more than one occasion to observe that a much larger comparative proportion of carbonic acid gas has been found mixed with the lower strata of air, than with the upper:" it were to be wished, however that he had substituted an accurate account of his experiments (and, especially, stated the temperature of the room at the time they were made), for this somewhat vague statement, more particularly as it clashes to a certain extent with the opinion of one of the most practised toxicologists of the day—M. Devergie, of Paris. (*Brit. and For. Med. Rev.*, Vol. VI. p. 243.)

There is a strange difference in the results obtained by different observers, regarding an apparently very simple enquiry, the proportion in which carbonic acid renders atmospheric air unfit to support combustion. Cavendish found that a wax candle was instantly extinguished in a receiver containing 11 per cent. of the gas; Mr. Taylor, that a taper burnt very well in air containing $\frac{1}{10}$ of carbonic acid, and was slowly extinguished when the bulk of gas amounted to 25 per cent.; whilst Dr. Ure fixed 15, and Turner 20 per cent. as the proportion capable of extinguishing a candle. These discrepancies, Dr. Bird has no doubt, may be traced to the differences in the diameter of the receiver used and in the kind of candle employed: the thinner the taper, according to this gentleman, and narrower the receiver, the more readily, *cæteris paribus*, does the former become extinguished. His experiments induce him to consider the statements of Turner and Mr. Taylor the most generally accurate. The practical value of such experiments is however materially lessened by the ascertained fact, that a candle will burn in an atmosphere containing such a proportion of carbonic acid as will prove rapidly fatal to animals: and, on the other hand, it is affirmed, that an individual has remained with impunity for several minutes in the atmosphere of a coal pit, in which ignited candles were repeatedly extinguished.

Dr. Bird's paper is illustrated by three cases: one already published by Mr. Chapman in the Lancet for December, 1838; another in which death was produced "by the inhalation of air containing carbonic acid evolved from a lime kiln;" a third, the notorious case of Trickey, who met his death in the year 1838 in St. Michael's Church, Cornhill. A report of the last by Mr. Gear had already appeared in the Lancet; the only important difference in the former and present versions being that, according to Mr. Gear, the deceased had voided his feces and urine, while no mention is made of his having vomited, immediately before death; whereas in the account transcribed by Dr. Bird from the notes of another practitioner, vomited food is said to have been found near the mouth of the deceased, but the contents of the rectum and bladder are not alluded to. It is needless to refer at any length to this case, which has already furnished the material for prolonged debate elsewhere.

The reader will observe that Dr. Bird, in ranging the second case in the same category with the others, carries into practice his assumption that carbonic acid is the toxic agent of charcoal vapour.

The most useful portion of Dr. Bird's production undoubtedly consists in two tables, exhibiting the most remarkable external appearances, as well as the results of dissection, of a certain number of individuals killed by carbonaceous fumes. The almost invariable presence of meningeal or cerebral lesions or both, is clearly demonstrated by these tables; and in the legitimacy of the following conclusions, drawn from them by our author, we are disposed to acquiesce, provided the words *charcoal vapour* be everywhere substituted for *carbonic acid.*

"1. Carbonic acid gas, sufficiently diluted with air (as in charcoal vapour), does not act fatally, by closing the glottis spasmodically, or by excluding oxygen, but as a specific poison. 2. Such an atmosphere will produce death, although it may contain a sufficient amount of oxygen to support life *per se*, and to allow the arterialization of the blood to proceed; on which account no dependence can be placed on the dark or florid colour of the blood, as arguments for or against poisoning by carbonic acid gas. 3. Such diluted carbonic acid acts most probably on the nervous system primarily; and, secondarily, by no means essentially on the circulating fluid. 4. The death of persons inhaling an atmosphere, vitiated by carbonic acid, is produced by the accession of apoplexy, often attended by serous effusion into the ventricles or on the surface of the brain, and occasionally even by extravasation of blood from some cerebral vessel. 5. No dependence can be placed on the bloated and red, or pale and contracted features; on the liquidity or coagulated state of the blood; on the injection or paleness of the mucous membrane of the intestinal tube or air-pipes; or on the flexibility or rigidity of the limbs, as positive arguments for or against the action of carbonic acid as a cause of death."

v. *Two Cases of Poisoning by the Inhalation of Carburetted Hydrogen.* By Mr. J. P. Teale.—These cases are essentially deficient, inasmuch as the subjects of them were not seen during life either by medical practitioners or even by nonprofessional persons; nevertheless, as the post-mortem examination was well conducted, and from the appearances thereby disclosed, as well as the circumstances under which death took place, no doubt can be entertained that the individuals perished from the inhalation of coal gas, Mr. Teale's paper is not an unimportant contribution to the pathology of gaseous poisoning.

The sufferers, Mrs. B. æt. sixty, very corpulent, and long subject to sudden attacks of loss of muscular power, and Miss W. æt. twenty-two, active and enjoying good health, occupied three small rooms in an alms-house at Leeds. On the 21st December, an explosion took place in one of these rooms on the introduction of a light, and removed a disagreeable effluvium of coal-gas, of which the inmates had complained during the day. At half-past ten both females retired to the same bed, and the following morning were found stretched upon it as if asleep, the bed-clothes appeared scarcely at all disturbed. On the arrival of some medical gentleman, the body of Mrs. B. was found to be quite cold, and the whole muscular system rigid; Miss W. appeared lifeless, but the trunk and extremities were warm, artificial respiration was accordingly performed, but without producing any indication of vitality.

A rent of about two inches in length was discovered in an adjoining gas-pipe, from which there was a ready transit for the effused gas to the rooms occupied by the deceased.

The dissection was made at half-past six the same evening: for the full particulars we must refer to the original paper, and confine our notice to the following appearances observed in both bodies in common: 1. A pallid appearance of the integuments and of the internal tissues, with the exception of some portion of the mucous membranes. 2. Florid discolorations on the neck and back. 3. Florid tint of the muscles. 4. Absence of venous congestion. 5. Fluidity of the blood. 6. Florid colour of the same fluid. 7. Infiltration of the lungs. 8. Injection and ecchymosis of the mucous membrane of the small intestines, and injection of the mucous membrane of the air passages. 9. The early occurrence of rigidity of the muscles after death.

In 1830, M. Devergie published an interesting account of poisoning with coal-gas in the Annales d'Hygiène. In this instance five individuals suffered more or less from its inhalation, and one of the number perished. As it is possible, however, that in the fatal case, a bean, discovered in the right bronchus, may have entered that tube before death, and so modified in some important particulars the post-mortem appearances, we shall not allude to them further than to say that the same pallor of the tissues, observed by Mr. Teale, existed also in the subject opened by M. Devergie. The latter pathologist noticed in his case a particular earthy discoloration of the liver, and seems inclined to invest such a condition with considerable importance as an anatomical sign of the poisoning under examination; our countryman's cases prove him to have been too precipitate in his inference. The prominent symptoms manifested by his patients were general prostration, marked debility, and somnolence, and the conclusion drawn by him from these and the morbid appearances is that coal-gas exercises a special deleterious influence on the economy, that it modifies the nature of the blood, and acts principally on the brain and liver. He also informs us that it produces its toxic effects, when it constitutes less than $\frac{1}{11}$ of the atmosphere; for a lamp remained burning all the night the accident occurred; and when the inmates of the house were roused, several candles were lighted without any explosion occurring.

The reader will derive instruction from a close comparison of the French and English contributions on the subject.*

VI. *Case of Imperforate Uterus, with Remarks.* By Mr. A. TWEEDIE.—Mr. Tweedie continues the history of a case of presumed imperforation of the uterus, in which delivery was effected through an opening made by incision, in the supposed situation of the occluded os. The operation with its immediate results were described in the second volume of the Reports, and briefly noticed in one of our early Numbers. The patient, Mrs. P., was again seized with labour-pains on the 31st of last December: on examination, Mr. Tweedie discovered an irregular opening, about as large as a penny, at the uterine extremity of the vagina, bounded anteriorly by a strong, firm, unyielding ridge, on which the cicatrix of the original incision was plainly felt. On the evening of the third day after

* See also Ollivier, Annales d'Hygiène, 1838, t. xx., p. 120.

labour had commenced (the pains had been urgent and energetic for about fourteen hours), no enlargement of the orifice having taken place since the morning, the pulse becoming quick, the skin and the vagina hot and dry, Mr. Tweedie deemed it unadvisable to wait longer, and, accordingly, made an incision, an inch long, through the cicatrix. Three quarters of an hour after the operation, the head was forced through the uterus, an additional rent of that organ having, it is believed, taken place, and the delivery was completed without further trouble. Three weeks after, the woman was able to walk a distance of four miles.

VII. *On Incision, in Cases of Occlusion and Rigidity of the Uterus.* By Dr. Ashwell.—Dr. Ashwell comments at some length on the case just referred to, and on a number of others of a similar character recorded by different writers.

It is manifest that, in cases which have been reported as examples of an imperforate condition of the uterus, the real state of the parts may have been either of the three following: there may have been total occlusion of the cervical orifice; or the os tincæ may have been shut out from all communication with the cavity of the vagina by adhesion of the posterior wall of the latter to the anterior surface of the uterus; or the womb may have been in possession of a normal inferior opening, tilted upwards and backwards beyond the reach of the finger of the accoucheur, in consequence of the organ being in a state of anterior obliquity. The difficulty of Mrs. P.'s first labour is referred by Mr. Tweedie and Dr. Ashwell to the presence of the first of these conditions; but no actual proof of the fact has been adduced, and it is purely hypothetical to consider the smooth attenuated part of the organ, where Dr. Ashwell practised his incision, as the site of the os closed up with adventitious structure. An example of the second condition was long since recorded by Gauthier,* in which that practitioner divided the anterior wall of the uterus by a transverse incision from right to left, and delivered his patient of a healthy full-grown infant with the forceps. A case of very similar character is related in Mr. Tweedie's paper from the practice of Dr. Hamilton. In these instances, as might be foreseen, one of the most remarkable peculiarities in the anatomical condition of the parts, was the shallowness of the vagina at its posterior aspect. The same anomaly existed in Mrs. P.'s case; and this, coupled with the fact that no cervix could be discovered, seems to lend some plausibility to the supposition that the orifice may have been excluded from the cavity of the vagina by adhesions of the latter to the uterus. But, as the reader is no doubt aware, the majority of reputed instances of imperforate uterus should,—if we be disposed to agree with a very numerous body of practitioners, among whom we may mention Desormeaux, Velpeau, Denman, Dewees, and Baudelocque,—be classed with our third category; the difficulty of finding a uterine orifice having, according to them, depended not on its deficiency from occlusion, but on its abnormal position. Now the fact, that from the time of Mrs. P.'s marriage up to the occurrence of labour-pains, "she had not a day's ill health," seems to militate in favour of

* Nouveau Journal de Médecine, t. vii., p. 36.

her case being one of persistent anterior obliquity, for it is hardly probable either that adhesion of the vagina to the cervix or conglutination of the orifice could have taken place without some local suffering and general reaction.

Dr. Ashwell dissents from the opinion of the above-named obstetricians, chiefly because "he has never met with any seriously protracted labours from obliquity." But if, not to speak of the experience of others, Mrs. P.'s uterus, at the period of her first labour, had really been affected with anterior obliquity,—and where is the conclusive evidence that such was not the fact?—Dr. Ashwell has most indubitably encountered at least one case of obstructed labour depending on that malposition; hence, in the assertion quoted, this writer has fallen into a palpable *petitio principii.* He *thinks* he could mention very respectable authors, whose experience corresponds with his own in this particular; if so, we cannot comprehend the advantage gained by concealing their names. He further alleges that, allowing the hypothesis of obliquity its full weight, it must be recollected, every hour of urgent uterine effort tends to rectify the displacement, and that if, after really powerful pains have been in action for ten or twelve hours, the os uteri be still undiscoverable, the legitimate inference is that no orifice really exists. We cannot subscribe to the justness of this argument, except its application be confined to cases of very trifling obliquity; unless the efforts of the patient be assisted by simultaneous attempts at reposition on the part of the accoucheur, whether the uterus be in a state of simple *anteversion* or of *anteflexion*, (in the latter case the os tincæ is of course within reach, but the progress of labour is not the less interfered with by the malposition of the body of the organ,) the play of the diaphragm and muscles tends to counteract the influence of uterine action, and augment the obliquity. In a case of this kind, in which no progress had been made after several hours' continuance of powerful pains, the first care of M. Velpeau, when summoned to the patient's assistance, was to direct her to moderate the violence of her efforts as far as possible: had he participated in the opinion expressed by Dr. Ashwell, his advice would have been widely different.

We must not omit to state that there is one very strong argument in favour of Dr. Ashwell's opinion, that Mrs. P.'s was originally a case of conglutinated os uteri. It seems, in truth, hardly possible that an artificial aperture in the body of the uterus could have remained open for a year, and that, if such an extraordinary occurrence had taken place, the foramen could have borne a sufficiently strong resemblance to the natural orifice, to be mistaken for that opening by the accoucheurs, who attended the woman in her second confinement. Extreme obliquity of the uterus is, besides, stated to occur rarely in primiparous women; this is, however, a point of inferior importance. The fact that Dr. Ashwell himself seems to have conceived no doubt of the reality of occlusion, carries considerable weight with it in our minds; for though in our character of reviewers we freely criticise his opinions, we have long learned to entertain the highest respect for that gentleman's scientific and practical acquirements.

But whatever be the cause of the non-expulsion of the fœtal head from the uterus, persistent obliquity, adhesion of the vagina round the cervix, or occlusion of the os, the cases become, practically speaking, identical, after the efforts of nature, aided by medical means, have been allowed to act unavailingly for a certain time. The principle on which relief is afforded is one and the same, an artificial opening must be made in order to lessen the chance of extensive and irregular laceration of the womb. The determination of the time at which this decisive measure should be had resource to, is, perhaps, one of the most difficult points in practical midwifery: Dr. Ashwell's directions for guiding our conduct are judicious, and to these we beg to refer the reader. A longer trial should be given to the natural efforts, assisted by attempts at reposition, in cases where the obstruction depends more on obliquity than on the other causes mentioned; but no reasonable argument can be adduced against the propriety of incising, when the malposition is found to be unchangeable, and, as in a case recorded by Lauverjat, the anterior wall is forced down to the genital fissure by each pain, at the imminent risk of rupture. In the case referred to, the operation was successfully practised, though *superficial* laceration of the substance of the uterus had already taken place. Instances of obliquity, in which such a measure becomes really called for, are, we hasten to add, extraordinarily rare.*

We have already described the practice of Gauthier in a case belonging to the second class: Dr. Hamilton, in the analogous instance referred to, "opened completely *the upper part of the vagina*;" this, we presume, means the portion of the uterus bounding that canal superiorly. The Professor does not inform his correspondent (the case is related in a letter to Mr. Tweedie,) whether his incision was transverse, antero-posterior, or crucial. From a fortnight after the lady's delivery, till she had perfectly recovered, and this is a point worth attending to, a dipt candle was, by his directions, daily introduced into the orifice.

In cases of occlusion, the practice is either to break up the adhesions with the finger, a bougie, or catheter, or divide them with a bistoury, and carry the incision to a certain extent beyond the lips of the orifice. Nägele, junior, is the advocate of the former proceeding; and where the occluding tissue is thin and fragile, it is evidently the most judicious,—provided also the surrounding substance of the uterus be healthy, and capable of normal dilatation.† But Dr. Ashwell justly observes, that Nägele's method of procedure is inapplicable, when the substance blocking up the os is thoroughly and firmly organized: admitting that such adhesion might be broken up by forcible traction with the finger, it would be, to say the least, imprudent to resort to the violent manipulation necessary for the purpose; the resulting contusion of the parts would be likely, by the local or general inflammation it engendered, to diminish materially the probabilities of recovery. We believe, too, with Dr. Ashwell, that

* Lauverjat reports another case of extreme anterior obliquity, in which bleeding, baths, and "other ordinary means" were employed, without producing any marked benefit. After sixty hours' continuance of pains, the orifice was sufficiently dilated to allow of the anterior border being reached with the finger; labour now advanced pretty quickly, but the infant was expelled in a putrid state; inflammation and gangrene of the uterus carried off the mother. The question is whether an operation, timeously performed, would or would not have averted this disastrous result. See *Nouvelle Méthode de pratiquer l'Operation Césarienne*, p. 72.—Paris, 1788.

† A case, successfully treated in this manner by M. Felix Hatin, has been recently reported in the French Journals. See p. 263 of our present Volume.

"it may, perhaps, be fairly assumed, that the risk of unlimited laceration of the uterus and adjacent parts, is much less, where incisions of tolerable extent have been discreetly made, than where merely a diminutive central aperture has been formed by a blunt instrument."

Our author next proceeds to consider those cases of much more frequent occurrence (such as that of Mrs. P. in her second labour, admitting that hers was in the first instance a case of *occlusion*), in which a *contracted os uteri* coexists with extreme rigidity, depending on organic disease of the surrounding tissue. He attempts to prove the general impropriety of persisting, for any length of time, in forcible attempts to dilate the orifice with the fingers, in cases of this description; and certainly succeeds in showing, from the recorded experience of others, that such dilatation is far from being the same non-hazardous and effectual operation here, that it is known to be when rigidity is unassociated with structural change. Regarding the precise moment when the operation should be performed, Dr. Ashwell observes, that *time* cannot be the sole element for regulating our interference; we ought not to hesitate about operating, "if the violence and frequent returns of the uterine efforts threaten rupture of the womb,—if there be distressing and constant pain about the neck or body of the uterus, or in any other part,—if the countenance becomes turgid and dark,—if perspiration issues at every pore,—if the pulse is full, strong, quick, and incompressible, and all this continue in spite of free venesection, and the exhibition of tartarized antimony, with or without opium."

There is a curious case transferred to our Sixth Volume (p. 235), from the Medicinische Zeitung, showing that the obstruction caused by a disorder of a different description, may require similar treatment for its relief. Here a large portion of the uterus prolapsed between the thighs, and all attempts to dilate the orifice to a greater diameter than that of half a dollar proving fruitless, an incision three inches in length was made in one side of the os, about thirty-six hours after the commencement of labour. Similar cases, terminating like it by the recovery of the mother, are related by Chopart and Capuron.* In two of these instances, it is not distinctly stated that diseased induration, beyond the general hardening consequent on the exposure of the lower portion of the organ to the air, and abnormal friction, existed in the cervix; in one of them, indeed, the close constriction exercised by the vagina on the neck, seems to have constituted the main hinderance to dilatation; in the other, the cervix was hard and callous. There are a few more such cases on record. The direction and length of the incision are obviously points of importance. In Mrs. P.'s case (her first labour), Dr. Ashwell carried his incision an inch forwards towards the bladder, and the same extent backwards to the sacrum: the preference appears to have been given to the antero-posterior incision, from apprehension of wounding the branches of the uterine arteries; no such accident, however, occurred in Gauthier's case, in which transverse division was practised. It is scarcely necessary to add, that the rectum and bladder should be evacuated before the incision is attempted; the other points of manipulation are such as must occur to every individual experienced in the surgery of the female genital

* Vide Churchill, Diseases of Females, p. 284.

organs. The use of the speculum, as originally had recourse to by Dr. Simson, is certainly not called for. Dr. Ashwell is, on *à priori* grounds, favorable to a crucial incision, as likely to prevent extensive laceration more effectually than the simple; we are disposed to coincide with him in his predilection. There is seldom much blood lost: should collapse and fainting occur, ammonia and brandy may be administered. The forceps has, in the majority of cases, been required for the termination of the labour.

Before quitting this subject we would, with Dr. Ashwell, enter our strongest protest against rash employment of the knife; the medical attendant should never have recourse to it, unless upon the deliberate conviction that his patient's safety cannot be otherwise secured; and to strengthen him in this conviction, as well as to shield himself from undue responsibility, the sanction of another practitioner should always be obtained.

VIII. *Observations on Fibrinous Concretions in the Heart.* By Dr. Hughes.—Dr. Hughes, like his predecessors, divides cardiac concretions, in respect of their cause, into two classes,—accumulations of fibrine resulting from retardation of the blood, and from inflammation of the endocardium. He conceives that the presence of the following characters in concretions, renders the fact of their formation, previous to death, incontrovertible; though he by no means affirms that the absence of these is conclusive of the cadaveric origin of the bodies so deficient.

"1st. When strict adhesion exists between them and the plane surfaces of the heart; and particularly when the membrane, from which they are detached, is found rough, vascular, and sprinkled with bloody points. 2dly. When a firm, white, fibrinous mass is found in one of the cavities, entirely separate, and detached from a dark purple, or mixed coagulum, filling up the remaining portion of the same cavity. 3dly. When, after the removal of the easily separable coagulated blood, we leave behind a smooth unbroken layer, or coating of fibrine, attached to a portion or the whole of the cavity in which that coagulated blood was previously lodged; though the layer or coating may be adherent, simply by passing behind and mixing with the musculi pectinati of that part of the organ to which it is attached. 4thly. When any changes, vital or chemical, the result of organization or degeneration, are observed in the concretion, which are not to be discovered in any other coagula existing in the same heart."

Some of the principles on which these propositions are founded are not so obviously correct as the author takes for granted; but a discussion of their merits would occupy more space than we can afford for the purpose,—we must, therefore, pass on to Dr. Hughes's description of his four varieties of concretions, caused by retardation of the blood, the *polypoid*, the *massive*, the *parietal*, and the *globular*.

The *polypoid* variety usually occurs in the form of a solid rounded mass of fibrine, varying in size from that of a filbert to a pullet's egg; it is sometimes attached by a broad base, at others by a narrow pedicle to the surface, and is often covered with a delicate membrane, capable of being injected. Its internal structure varies with its age, and the degree of decomposition it may have undergone.

The *massive* is of exceedingly irregular shape, from the number of prolongations arising from its surface, and passing into the depressions, or through the orifices, of the cavity in which it is placed. There is often much difficulty felt in determining its date, but when most decidedly antecadaveric, it is thin and expanded, of a pinkish white colour, firm in texture, easily separable into layers, and attached to some of the plain surfaces of the heart by simple adhesion. The adhesion is, however, principally dependent on the intertwining of the processes alluded to among the musculi pectinati, or chordæ tendineæ, and the prolongation of the foreign body into other cavities, whereby the channel for the passage of the blood is considerably narrowed.

Of the *parietal* species, Dr. Hughes has seen only one recent example; this formed a general lining for the cavity in which it was found, and consisted of an irregular sort of network, about a line thick and two broad, firm in point of consistence, and with a smooth glistening surface.

The *globular* concretion varies in size, from that of a pea to that of a pullet's egg, is of an opaque white, or light dirty brown colour, smooth and even externally; when large, cystiform, and containing fluid. In the latter case, the wall of the cyst is seldom more than a line in thickness, but separable into layers; the contained fluid consists of blood, either healthy or in an abnormal state; of serum, or of a fluid which bears a certain degree of resemblance to pus: of this, more by and bye. These concretions are either with or without pedicles. When situated in the appendices of the auricles, they affect different characters from those we have enumerated; for these we must refer the reader to Dr. Hughes's pages.

Dr. Hughes illustrates the relative frequency with which the different cavities contain fibrinous concretions, by a tabular view of sixty-two cases, collected from various sources. From these we learn the following proportional numbers.

32 on the right side of the heart.
38 on the left.
15 in the right auricle.
21 in the right ventricle.
14 in the left auricle.
27 in the left ventricle.
7 in the right and left sides at the same time:
4 in the right auricle and ventricle.
3 in the left auricle and ventricle.
2 in both auricles.
6 in both ventricles.
1 in all the cavities.
1 in the right auricle and ventricle, and left ventricle.
1 in the right auricle and left ventricle.

These numbers seem to show that the opinion generally held by authors,—at least Dr. Hughes ascribes such opinion to them,—namely, that these bodies are most frequently met with on the right side of the heart, is incorrect. M. Bouillaud does, we think, make an assertion to the effect stated by our author, but he evidently intends it to refer to concretions of all kinds, and not simply to those caused by retardation of the blood. Now had Dr. Hughes included in his table the forty-nine cases of cardiac concretion scattered through the French writer's volumes, and not simply reckoned the six examples given in the chapter especially devoted to the subject, he might have still found the common opinion to be substantially correct.

Dr. Hughes proceeds to speculate on the mode of formation of his first class of concretions. We differ with him respecting the alleged peculiar proneness of the blood to coagulation, in long-standing diseases of the heart; and when, in attempting to account for the production of certain concretions, assumed to belong to this category, he has recourse to the influence of an inflamed endocardium—even though that influence be an indirect one—he forgets that the fact of such causation entitles them to be ranked with his second order.

No new hints are thrown out for determining the exact age of these concretions. Respecting the changes occurring in the globular species of the bodies under consideration, and the nature of their central fluid, Dr. Hughes, after enumerating the principal hypotheses brought forward in explanation of the presence of a puriform liquid in their interior, declares his own persuasion to be, that such liquid is never composed of true pus. M. Magendie states, in the first of his lectures on the blood, already reviewed by us, that in one particular instance he found that the fluid, contained in a fibrinous sac entangled in the walls of the right ventricle, was not composed of pus, but of tuberculous matter: this observation (which, strange to say, he quotes incorrectly,) is adduced by our author as confirmation of the opinion he holds; we confess we do not share in his belief of its importance. Be this as it may, Dr. Hughes conceives that the formation of the variously-coloured fluid may be "simply explained" as follows. "A concretion containing many, few, or no red particles, according to the celerity or tardiness with which it originally assumed, exteriorly, a solid form, and more or less serum in proportion to the amount of subsequent contraction, may be incapable of organization. Subject, therefore, to the laws of unorganized or unorganizable matter, that portion containing the most serum, or first coagulated, gradually softens and breaks down, or degenerates into a fluid, the physical characters of which depend on the materials of which the concretion was originally composed." So far as we can perceive, this theory is nothing more than a somewhat pompous enunciation of the doctrine of "softening," "breaking down," or "decomposition" of fibrine, advocated by Dr. G. Burrowes, by Dupuytren, and recently by Mr. G. Gulliver.*

On fibrinous concretions induced by and complicating endocarditis, Dr. Hughes makes no observation of novelty,—except that, when attached to the surfaces of the valves, bodies of this class are uniformly found on the side opposed to the direct current of the blood.

The section devoted to the symptomatology of cardiac concretions, is neither full nor satisfactory; Dr. Hughes appears to have had very limited clinical experience on the subject. He suggests, on the authority of one of M. Magendie's recently broached crotchets, the administration of the salts of potass and soda, in the hope of dissolving such portions of fibrine as may have been deposited in a concrete state, and of decreasing the coagulability of the remainder.

Among our Foreign Selections will be found an analysis of a paper on the same subject, by M. Bouillaud, which may be regarded as the complement of the present essay.

* Medical Gazette, March, 1839.

IX. *Analysis of Bones affected with Mollities Ossium.* By Dr. Rees.—Dr. Rees has analyzed those specimens of bone affected with mollities, in order to ascertain whether the law, observed to regulate the relative proportions of animal and earthy matter in the healthy tissue of different portions of the skeleton, does or does not hold good in respect of bones so diseased. The bones examined were the fibula, one of the ribs, and a vertebra; and it will be perceived, from the subjoined table, that in the morbid as well as in the natural condition, the first-named bone contains more earthy salts than either of the others, and the second more than the third.

| | Mollities. | | Health. | |
|---|---|---|---|---|
| | Earths. | Animal Matter. | Earths. | Animal Matter. |
| Fibula - - | 32·50 | 67·00 | 60·02 | 39·98 |
| Rib - - | 30·00 | 70·00 | 57·49 | 42·51 |
| Vertebra - - | 26·13 | 73·87 | 57·42 | 42·58 |

X. *Case of Division of the Tibia for the Cure of Deformity, occasioned by a Gunshot Wound.* By Mr. Key.—This is a case of very marked deformity of the right leg, resulting from the loss of a considerable portion of the inner surface of the tibia, accompanied with fracture. The injury, caused by a musket-ball, occurred in the East Indies, in August, 1835. Examined by Mr. Key, in October, 1838, the broken ends of the tibia were found to have united at an angle, and the bone to have deviated from its natural line, in relation to the femur. The head of the fibula had, besides, been forced away from its articulation with the tibia, and formed an unnatural prominence above the usual position, in reference to the last-named bone. As the fibula was not broken at the time of the accident, it maintained its natural straight figure, the direction of the whole bone being parallel to that of the lower division of the tibia. The limb was shortened to such a degree, as to oblige the patient to walk on his toes; and the heel raised an inch and a half from the ground, when he stood upright. It was decided, in consultation with Sir Astley Cooper, that the limb might be restored to a useful state, and the deformity remedied. On the 14th of October this decision was acted on by Mr. Key. This gentleman sawed the tibia across, in the situation of the fracture, just below the attachment of the soleus muscle, and found, that by this operation, the lower part of the leg became capable of being freely moved in every direction; there was consequently no necessity, as it was apprehended there might be, for dividing the fibula. The limb was allowed to rest on pillows, without much restraint, for about ten days after the operation; at the end of this period, the wound had gone through its several stages, without exciting more than inconsiderable constitutional disturbance. The limb was thenceforth kept straight, with an under and two lateral splints, well padded,—a tourniquet being applied at either end of the splints, as it was found that the constant tendency to displacement could not be effectually prevented by common tapes and bandages. The process of union and consolidation was necessarily tedious, from the limited extent of contact between the corresponding surfaces of the tibia; at the beginning of January last, union had, however, taken place, and on the 24th of the same month, the patient was able to leave town. On the

10th of March, the long splints were discontinued, and the leg maintained as good a position as when the patient had been in town. The cicatrization of the wound had been retarded, by the exfoliation of several pieces of bone; in other respects, the patient was going on well.

This operation reflects the highest credit on the distinguished surgeon who performed it. The change effected in the bearing of the leg is well shown in an engraving.

XI. *Case of Spermatocele, or Varicocele, treated by Excision of a Portion of the Scrotum.* By Mr. Bransby Cooper.—We have here a case of varicocele (treated by excision of a portion of the scrotum) which is, according to the narrator, as well as the inventor of this operation, a most favorable instance of its successful adoption. The perusal of Mr. Bransby Cooper's narrative convinces us, notwithstanding, that the new operation is scarcely, if at all, less perilous than Professor Breschet's method, of obliterating the dilated veins by instrumental pressure.* Mr. Bransby Cooper dreads phlebitis, and finds fault with former modes of operating, on the score either of their acknowledged proneness to bring on, or of their being actually designed to produce, that inflammation. He is perfectly correct, in looking on the dangers of phlebitis as the main objection to their general employment. But that a similar inconvenience appertains to the present operation is abundantly evident, from the details of his own case; nay, more, that Mr. Cooper himself considers phlebitis to be a necessary element in ensuring a successful issue, appears from his own conception of the *modus operandi* of the novel procedure. To the truth of this, his own words shall bear witness:—"It appears to me, however, and indeed seems apparent, from the daily report of the above related case, that the excision of the portion of the scrotum leads to the cure of spermatocele, *by inducing inflammation, and consequent obliteration of the diseased veins;* and without the same risk as attends upon the application of any immediate means to the veins themselves, as must be the case, either by the employment of a ligature, or the excision of the varix." It will be perceived that Mr. Cooper differs in his notions, respecting the manner in which the cure is effected, from his gifted relative. Sir Astley believes the success of the operation depends on the constriction exercised on the veins, in consequence of the reduced bulk of the scrotum. Mr. Cooper's assertion, that phlebitis, produced in the present manner, is less likely to prove dangerous than the same disease when otherwise caused, is, we need hardly point out to the reader, purely hypothetical.

XII. *Observations on Abdominal Tumours, and Intumescence: illustrated by Cases of Renal Disease.* By Dr. Bright.—The contents of this valuable contribution to renal pathology will be noticed elsewhere.

* Vide Walshe on Breschet's operation for varicocele, in *Medical Gazette*, Dec. 1834; and *British and Foreign Medical Review*, Vol. VIII. p. 254.

No. IX.

I. *On the Dislocation of the Os Humeri upon the Dorsum Scapulæ, and upon Fractures near the Shoulder Joint.* By Sir Astley Cooper.—This communication opens with an account of a doubly remarkable luxation of the humerus into the infra-spinata fossa. In the first place, the displacement occurred during the convulsive struggles of an epileptic fit, at a time when the patient was watched by his wife, who avers that he neither fell from his bed, nor dashed his shoulder against any resisting surface; and secondly, the head of the humerus could, by extension, be drawn into the glenoid cavity, but the moment the traction was discontinued, the extremity of the bone slipped back, with a grating sound, on the dorsum scapulæ. The attendant surgeons felt persuaded, that the mobility of the head of the humerus depended on a piece of the rim of the glenoid cavity, or of the lesser tuberosity, or of the head itself, having been broken off. The incorrectness of this notion was, however, established on the patient's death, seven years after the occurrence of the dislocation, which had never been reduced; no trace of fracture could be discovered. "But the tendon of the subscapularis, and the capsular ligament, had been torn from the smaller tubercle of the os humeri, and the bone was consequently drawn back by the action of the infra-spinatus and teres minor muscles, there was no support given to the head of the bone, when returned into its cavity: but so soon as it was replaced, it was drawn back by the action of the two posterior muscles, and all the appearances of dislocation reproduced." "Under these circumstances," adds Sir Astley, "the bandage, required to keep the bone in its place, should be placed on the fore part of the chest, and behind the shoulders, to keep the head of the bone forwards; and a pad ought to be placed behind the head of the bone, to prevent it from slipping from its cavity; . . . . . to this bandage a sling must be added, to support the elbow, and to keep it back." The reader will do well to consult the description and engraving of the ingenious apparatus, constructed on this principle, and employed by M. Sédillot in a case of dislocation into the infra-spinata fossa, reduced by that gentleman *a year and a fortnight* after its occurrence.*

Sir Astley next proceeds to examine the diagnostic and anatomical characters of certain accidental lesions, which are liable to be confounded with simple dislocation. He reduces these to three classes: 1. Dislocations forwards, with fracture, and detachment of the head of the bone. 2. Fractures through the anatomical neck of the humerus. 3. Fractures through the surgical neck. We need only thus present the reader with the bill of fare, and the name of the master of the feast, to induce him to become a guest at the board.

II. *Case of Poisoning by Sulphuric Acid.* By Mr. A. Taylor.—There is nothing particularly remarkable in the history of the patient here referred to, a man aged forty, who died twenty-five hours after swallowing a wine-glass full of sulphuric acid. The pharynx, œsophagus, and stomach, presented the usual evidences of the action of the acid; but

* Mémoire sur une luxation de l'épaule en arrière, &c. Paris, 1833.

not the slightest indication of free, or combined sulphuric acid, was obtained from the analysis of the contents or substance of the latter organ: the poison had been effectually removed by vomiting and purging, aided, perhaps, by the neutralizing action of magnesia, which had been freely exhibited. The aortic lining membrane was of a bright scarlet colour; but the vessel was filled with blood, and the body examined three days after death; hence, so far as Mr. Taylor's details go, they justify the conclusion, that the red tint resulted from imbibition. The state of the blood is not noted with sufficient precision to enable us to determine, whether the case furnishes evidence for or against M. Magendie's notions respecting the action of sulphuric acid on that fluid.

III. *On Amputation.* By Mr. Bransby Cooper.—Mr. Cooper, alarmed by the rapid extension of railways, and increasing employment of machinery, revives the *vexata questio* of the propriety of amputating, after certain severe injuries, or of attempting to save the limb by treatment. The enquiry into this point is rather confusedly mixed up with the examination of a very distinct problem: namely, the necessity of amputating being incontestable, what is the precise time, after the infliction of the injury, at which the operation should be performed? The latter point is thus decided: "when there can be no hope of saving the limb, its removal should be effected at the earliest opportunity, before inflammation and swelling have commenced, . . . . unless the patient be in a state of collapse; in this case, the surgeon must wait for reaction." Such is, if we mistake not, the doctrine inculcated by surgeons generally: Mr. Bransby Cooper brings forward no novel argument in its support.* In respect of the former, we find an admission, that it is very difficult to define the precise extent of injury requiring immediate amputation; of this, all who have simply witnessed the practice of a large hospital, without ever having visited the "tented field," will recognize the justness: our author's information is, unfortunately, not calculated to lessen the embarrassment of the surgeon called upon to decide such a question.

Before taking leave of this paper, we would notice the statement, that in three or four cases of want of union of fractured bone, depending on constitutional causes, Mr. B. Cooper "has found the administration of mercury, to an extent to produce ptyalism, of the greatest benefit; and has succeeded in producing consolidation, after seton, pressure, and other violent means had failed." It seems only necessary for its admirers to try this marvellous mineral in a new way, to ascertain its possession of a new virtue: it promotes absorption, or gives new energy to nutrition, lowers or excites vascular reaction, &c. &c., just at the will of the exhibitor; all depends on the *animus* with which it is given.

IV. *Cases of Hernia.* By Mr. Bransby Cooper.—The practical value of this paper atones for the somewhat unsatisfactory character of its predecessor. The patient referred to in the first case, aged sixty-eight, had for some years been the subject of right inguinal, and, for some months, of left inguinal hernia. He was suddenly seized

* The reader interested in the subject will find this point excellently discussed in the Cyclopædia of Practical Surgery. Art. Amputation, p. 129.

with abdominal pain, chiefly epigastric, not increased by pressure, and frequent vomiting, on the 26th of April; the bowels had been twice moved in the course of the day. Purgatives were ordered, and one stool procured the following morning; from this time, however, the symptoms of strangulation became progressively more evident; the patient had no further motion, the matters vomited acquired the quasi-fœcal aspect, and hiccup supervened, followed by death, in about eighty-four hours after the first symptoms were noticed. The inguinal regions had been attentively examined on the second day of the patient's illness, by Mr. Toulmin: the right, in which a truss had been worn for years, appeared unnaturally flat; in the left, a small hernia, reducible without the least difficulty, was easily discovered, and this side seemed otherwise unusually full, from an accumulation of fat. On the evening of the fourth day, Mr. Bransby Cooper found besides, by closer examination, that no suspicious condition of any kind existed in the inguinal region of the right side. The fullness of the left, was taken for evidence that the mischief lay in that direction; the inguinal canal was, in consequence, laid open, and found to contain a small empty sac, but nothing to account for the symptoms. The patient died the morning after the operation. On examination, a piece of intestine, as easily reducible as during life, was discovered in the sac on the right side; but, on attempting to draw the intestine out of the situation of the internal ring, some resistance was experienced, and, on enquiry, ascertained to depend on "a portion of intestine having become strangulated in a small hernial sac, situated anteriorly to the larger one, containing the reducible hernia." Now as the strangulated portion of intestine lay within the abdomen, it is manifest that an operation would not have led to the relief of the stricture, unless the large reducible hernia had been cut down on while within the scrotum. "It may be learned from this case," observes Mr. Cooper, "that when, after the reduction of a hernia by the taxis, the symptoms of strangulation remain unabated, the tumour should, if possible, be again made to protrude, and then be submitted to operation; as it is evident its reduction has not relieved the constriction of the implicated viscus." The general advisability of the practice here recommended, may not be very questionable; but as, under the circumstances supposed, no proof can be obtained that the obstruction resides in the portion of bowel connected with the sac, the surgeon must be exceedingly guarded in promising ultimate benefit from the proceeding. The case very strongly illustrates the propriety of examining into the exact state of a hernia, when the stricture has been divided external to the sac, before returning it into the cavity of the abdomen.

A second, and very similar case, which is next related, leads Mr. Cooper to the further conclusion, in respect of treatment, that if the hernia cannot be reproduced in the manner above referred to, "the outlet of the abdomen through which the protrusion had occurred, should be laid bare, and dilated, so as to facilitate the descent; or even, if necessary, so as to expose the hernial sac, and enable the operator to divide the stricture." The probable success of this manœuvre is less than that previously spoken of, as the operation is confessedly both dangerous and difficult,—nevertheless, we agree with Mr. Cooper, that it is better to give it a trial, than to consign a patient to inevitable dissolution. However, we should, most indubitably, first put into practice the mode of treatment introduced by Mr. O'Brien, and which has apparently led to very fortunate results. To this important addition to surgical therapeutics, Mr. Cooper, strangely enough, does not advert.

v. *Case of Empyema and Pneumothorax; with Observations.* By Dr. Barlow.—This is a case remarkably confirmative of the doctrine advanced by Drs. Houghton and Stokes, in opposition to the opinions of Andral and Louis, that perforation of the lung, in tuberculous subjects, is not only not necessarily mortal, but may actually prolong life, and arrest, for a time, the constitutional symptoms of phthisis.

Anna G——, aged twenty-one, previously enjoying good health, according to her statement, was seized about the middle of 1835,* with pain in the left side, dyspnœa, and short dry cough, no marked febrile attack being recollected; at the commencement of 1836, she became unable to lie on the left side, and was conscious of a sensation like the splashing of water within the left side of the thorax; subsequently, the dyspnœa became extreme, her strength suddenly failed, and she expectorated copiously, *for the first and only time.* Seen, ten days after, by Dr. Barlow, she was found lying on the right side, respiration very hurried, pulse 120—130, cold sweat, profuse diarrhœa; her voice was reduced to a mere whisper. The physical signs were, on the *left* side, considerable dulness under the clavicle, sound more natural a little lower down, preternaturally clear below the inferior angle of the scapula; no respiratory murmur; amphoric sound audible, on deep inspiration, below the axilla, and over a considerable part of the posterior surface of this side; splashing sound heard distinctly, on succession: on the *right* side, doubtful dulness under the clavicle, resonance elsewhere good; respiration imperfect in the former situation, puerile in the latter. The heart appeared to be in its natural situation. The following year the patient had improved so much as to be able to walk about, the sound of splashing ceased to be audible, but the amphoric respiration persisted; the diarrhœa and sweats had been completely controlled, the pulse had sunk to 70. From this period until that of her death, which occurred on the 26th of February, 1839, the patient's condition underwent some slight change for the worse each winter, with a corresponding improvement in the summer. At the beginning of 1838, when the splashing sound became again perceptible, the impulse of a fluid in the left side of the chest was felt, by placing one hand on the scapula and the other under the clavicle, and briskly shaking the patient; the upper part of this side was now found to be preternaturally resonant, and in the month of September dislocation of the heart, to the right side, was discovered.

The body was examined twenty-four hours after death. The *left* side of the chest, lined with false membrane coated with curdy matter, contained about a pint of inodorous pus; at the upper part of this cavity lay the superior lobe of the lung, about half as large as an egg, and composed almost wholly of truncated fragments of bronchial tubes (some of them with open mouths) and tuberculous matter. The lower lobe, non-tuberculous, was firmly compressed against the bodies of the vertebræ.

* Dr. Barlow's dates are very loosely stated.

There were universal old pleuritic adhesions on the *right* side, with cavities in the upper lobe; the middle and inferior lobes nearly filled with tubercles, or gray granulations. The heart and aorta were healthy, but small; the former situated to the right of the middle line.

Dr. Barlow conceives that the unusual continuance of life in this case resulted from the suppression of the disease in the left lung, its slow progress in the right, and the diminution of constitutional irritation, which occurred subsequently to perforation. There can be little doubt of the correctness of this notion, for it is hardly more than an expression of facts; but the same degree of certainty does not attach to the inference that all these fortunate circumstances were the direct consequence of the establishment of hydropneumothorax by perforation of the lung. In the first place, it is exceedingly hypothetical to suppose that the condition of the left lung could have any influence in retarding the deposition of tuberculous matter in the right. Dr. B. presumes, with no little ingenuity, that the secretion of pus in the pleura may have acted in respect of the opposite organ in the manner of external counter-irritants; but there is no proof even that this secretion was constantly or habitually going forward. On the other hand, that the deposition of tuberculous matter in the lower left lobe may have been prevented by the pressure of the effused fluid, is highly probable: herein, therefore, the intercurrent pleuritic disease was a favorable condition for the patient's recovery. Thirdly, the obliteration of the diseased lung is considered by Dr. Barlow, in imitation of Dr. Stokes,* to have acted on the constitutional state of the subject, as the removal by art of a scrofulous joint; the recovery of flesh, strength, and health, which occasionally follows the latter operation, needs not to be dwelt on. This is a shrewd and plausible conjecture of the Irish pathologist, and in the present case derives support from the fact, that the *right* lung was very slightly affected at the time perforation of the *left* supervened. A somewhat analogous occurrence is occasionally, we may add, observed in the instance of external carcinomatous growths: in a fair proportion of the cases in which removal of the morbid structure has been followed by permanent cure, Nature, by setting up gangrene in the surrounding tissues, and enucleating the foreign mass, has been the successful operator.

There is a peculiarity in the physical phenomena of Dr. Barlow's case, to which that gentleman does not allude. When careful examination was first instituted, the sign of the presence of air was well marked at the *lower part* of the chest, while considerable dulness prevailed at the anterior *summit;* nor does the existence of tympanitic sound in the latter position, appear to have been established until two years afterwards. This apparent infringement of the laws of gravitation may be explained, by supposing the tuberculated lung to have been adherent at its summit to the thoracic parietes in the outset, while its gradual destruction by the progress of the tuberculous disease slowly effected its separation therefrom, and permitted the circulation of air around it. But objections may, we are aware, be raised against this conjecture; Dr. Barlow's details, unluckily, do not supply materials for a better.

The proportion of cases in which perforation causes death, during the acute stage of the consequent pleuritis,—or, on the contrary, exercises

* Diseases of the Chest, p. 531.

no apparently baneful influence on the health of the patient, is a point to be decided by the application of the numerical method. M. Andral has materially exaggerated its fatality, while Dr. Stokes appears to have fallen into the opposite error. Can the existence of a third class of cases, in which actual benefit has apparently accrued to the patient from the effusion of air into the chest, be admitted as evidence that Dr. Carson's idea of treating phthisis by inducing artificial pneumothorax, is not quite so infatuated as has generally been believed?

VII. *On the Physical Diagnosis of Incipient Phthisis.* By Dr. HUGHES. —VIII. *Observations on the Pulse in Phthisis Pulmonalis.* By Dr. GUY.— We have noticed these papers in the First Article of the present Number.

IX. *On the supposed Existence of Fluoric Acid as an Ingredient in certain Animal Matters.* By Dr. REES.—Dr. Rees, continuing his sagacious enquiries into the chemical constitution of bone, has arrived at a result subversive of the commonly received notions, founded on the analyses of Gay Lussac and Berzelius, respecting the presence of fluoruret of calcium in that and other animal substances. He is firmly convinced that no such salt exists either in recent ivory, the enamel of teeth, human bone, or urine,—that it should be expunged from the list of constituents of animal compounds, and that its presence in fossil ivory is the result of partial mineralization of that substance. The error of the continental chemists, he conjectures, must have originated from their using glass of inferior quality as a test for the fluoride. It appears that on distilling equal parts of sulphuric acid and water on bone earth, till the measure of water passes over, the latter is found to contain a proportion of phosphoric acid, and that on evaporating the fluid, the acid partially volatilizes with it: now it is well known that phosphoric acid, if heated to volatilization on glass of *inferior quality,* will corrode it with considerable energy.

X. *Observations on the Incubated Egg.* By Mr. JOSEPH TOWNE.— Mr. Towne relates some ingenious experiments and observations on certain phenomena of incubation. He conceives he has demonstrated the fallacy of the chief element in the received theory of the decarbonization of blood in the chick,—namely, the passage of atmospheric air through the shell. His first experiments on this point were made by investing the shell with four coats of paper and albumen, and watching the subsequent development of the chick, which, as he avers, underwent no perceptible modification. Additional security against error from permeability of the investing substance was obtained by using "five thicknesses of stout paper, and then adding three thick coats of oil-paint, composed of white lead, with a large portion of sugar of lead used as a drier: this I did, with the double intention of offering an additional obstruction to the air, and also to prove whether the paper was, or was not, entirely sufficient for this purpose; concluding that, if it were not, the fume from so noxious an application must inevitably prevent the progress of incubation. All this, however, had no effect: I opened the egg at twelve and a half days, and found that no interruption had occurred, the venous and arterial circulation being perfectly natural."

Mr. Towne further concludes, from these experiments, that the air contained in the *folliculus aëris* is not used, as stated by Sir Everard Home, to aerate the embryo during the early stage of its development; they are, in this point of view, confirmed by the fact that the internal paries of the folliculus remained unruptured in the covered eggs. The following passage will allow the reader to judge of Mr. Towne's capability for physiological investigation.

"I next examined a number of eggs, to ascertain when the folliculus aëris becomes ruptured: and this is best done by dividing the shell with a sharp file, about one inch from the small end of the egg; because in this way it can be separated without fraction, and consequently without injury to the membranes: the chick may then be withdrawn, and the chorion removed with a pair of forceps: after which a large aperture should be made at the large end; and then, by gently blowing in, it will be evident whether the membrane be ruptured or not. I found this had not occurred on the eighteenth day; so that the air contained in this cavity would appear to have some other use than the one assigned to it by Sir Everard Home. I also observed, that up to this time the chick had not the power of using its vocal organs: but, on the nineteenth day, so soon as the forceps were introduced, it uttered a loud note of distress; and in this egg there was a large rupture in the membrane of the folliculus aëris. I believe, therefore, that the air which is contained within the egg is not rendered available for decarbonizing the blood until the nineteenth day: certain it is, that then the membrane is ruptured; and then it is that the chick first has the power of using its vocal organs. Now if it be considered, that at this period the chick has nearly reached maturity, and is soon to leave the shell in which it also leaves the chorion,—and that the large umbilical vessels, through which the blood has hitherto been conveyed to that membrane, are beginning to shrink, and have a dried appearance,—in short, that they are assuming a character which will allow of their division without loss of blood, and consequently are no longer capable of performing their original function—it will, I think, be sufficiently apparent why thus should be furnished a reservoir of air adequate to supply the lungs of the chick, until it has effected an opening through the shell."

We cannot continue this notice further than to add, that the remaining part of the paper exhibits much sagacity in the conception and interpretation of experiments; but however ingenious these may be, they obviously require to be repeated frequently, before the important conclusions now drawn from them can be looked on as fully established.

Some very exquisite coloured lithographs illustrate the text; and the models placed by Mr. Towne in the Museum at Guy's, representing the different stages of the process of incubation, are indeed *models* in their way.

XI. *Report of Primary Syphilitic Cases.* By Mr. Aston Key.—We postpone our examination of this paper, until its author has completed it.

XII. *On the Preservation of Subjects for Anatomical Purposes.* By Drs. Babington and Rees.—The object of this article is to recommend the injection of pyroxylic spirit as a preservative against the putrefaction of subjects intended for dissection. The special advantages of employing it are stated to be its extreme fluidity, its freedom from colour, its cheapness (it is however half as dear as alcohol), and its innocuous nature. The smell of subjects injected with this ingredient appears to be vastly disagreeable; a full description of it will be found in the Annals of Philosophy, New Series, viii. 69: it is perfectly distinct from pyroligneous acid and pyroacetic spirit.

